# Intratumoral Heterogeneity of MAGED4 Expression in Oral Squamous Cell Carcinoma: Epigenetic Mechanisms and Therapeutic Implications

**DOI:** 10.3390/ijms262411772

**Published:** 2025-12-05

**Authors:** Huan Xie, Feng Li, Xiaoqiong Zou, Xiaoqing Yu, Sheng Zhang, Yanjing Wang, Weixia Nong, Limin Xie, Yi Wang, Bin Luo, Xiaoxun Xie, Qingmei Zhang

**Affiliations:** 1 Department of Histology and Embryology, School of Basic Medicine Science, Guangxi Medical University, Nanning 530021, China; 202210001@sr.gxmu.edu.cn (H.X.); li-feng@mail.tsinghua.edu.cn (F.L.); zxq@sr.gxmu.edu.cn (X.Z.); 202320027@sr.gxmu.edu.cn (X.Y.); 202320030@sr.gxmu.edu.cn (S.Z.); 202020023@sr.gxmu.edu.cn (Y.W.); nwx@sr.gxmu.edu.cn (W.N.); 330056@sr.gxmu.edu.cn (B.L.); 2 General Practice School, Guangxi Medical University, Nanning 530021, China; 20235153669@sr.gxmu.edu.cn (L.X.); 20235153689@sr.gxmu.edu.cn (Y.W.); 3 Key Laboratory of Basic Research on Regional Diseases (Guangxi Medical University), Education Department of Guangxi Zhuang Autonomous Region, Nanning 530021, China; 4 Guangxi Health Commission Key Laboratory of Basic Research on Brain Function and Disease, Nanning 530021, China

**Keywords:** MAGED4, OSCC, intratumoral heterogeneity, DNA methylation, histone acetylation

## Abstract

Intratumoral heterogeneity poses significant challenges to the efficacy of cancer immunotherapy. Melanoma-associated antigen D4 (MAGED4) has been proposed as a potential immunotherapeutic target in oral squamous cell carcinoma (OSCC). This study aims to investigate the expression of MAGED4, focusing on its intratumoral expression heterogeneity and the underlying epigenetic regulation mechanism. Utilizing public online databases, immunohistochemical analyses of clinical specimens, and single-cell RNA sequencing data, we found that MAGED4 was overexpressed with significant intratumoral heterogeneity in OSCC tissues. Methylation-promoter luciferase reporter assays revealed that *MAGED4* transcription was suppressed by DNA methylation at its promoter region. Additionally, co-expression analysis implicated a potential role for histone acetylation in regulating *MAGED4*. To functionally validate these findings, we treated OSCC cells with the DNA methyltransferase inhibitor 5-aza-2′-deoxycytidine (DAC) and histone deacetylase inhibitors trichostatin A (TSA) and valproic acid (VPA). The triple-drug combination treatment resulted in the most robust reactivation of MAGED4 expression, correlating with promoter DNA demethylation and enhanced acetylation of H3K9 and H3K27 at the MAGED4 promoter. Our findings elucidate critical epigenetic mechanisms contributing to MAGED4 heterogeneity in OSCC and highlight the potential of combination epigenetic therapies to reverse this heterogeneity, thereby providing a foundation for exploring such approaches to improve immunotherapeutic outcomes.

## 1. Introduction

Oral squamous cell carcinoma (OSCC) is one of the most prevalent malignancies in the head and neck, ranking as the sixth most common cancer worldwide [1]. The standard treatment strategy involves radical surgery combined with adjuvant radiotherapy or chemotherapy. Despite continuous advancements in treatments, the 5-year survival rate for OSCC patients remains stagnant at approximately 40–50%, indicating a pressing need for improved prognostic outcomes [2]. Over the past decade, programmed death-ligand 1 (PD-L1) has emerged as a key target for cancer immunotherapy, demonstrating significant efficacy in various refractory tumors [3]. However, the efficacy of PD-L1 monotherapy in patients with OSCC remains notably limited [4]. Therefore, the development of effective early diagnostic biomarkers and novel combination therapeutic strategies has emerged as a critical and challenging research imperative.

Cancer immunotherapy has recently emerged as a promising approach for OSCC treatment, wherein the identification of tumor-specific antigens is a critical step. The melanoma-associated antigen (MAGE) gene family, comprising more than 60 members, has gained attention as a highly promising immunotherapeutic target due to its high expression in various malignancies and restricted expression in normal tissues [5]. Several clinical trials have demonstrated the therapeutic potential of certain MAGE members, such as MAGE-A1 and MAGE-A3, in solid tumors [6,7]. However, the expression patterns of different MAGE antigens vary significantly across tumor types. Thus, it is clinically important to identify the specific MAGE members applicable to oral cancer.

MAGED4, a member of the MAGE family, is typically characterized by absent or low expression in the majority of normal tissues [8,9], while being upregulated in OSCC tissues [10,11]. The overexpression of MAGED4 has been associated with enhanced tumor cell proliferation, migration and poorer patient prognosis [10]. Notably, antigenic peptides derived from MAGED4 have been found to elicit specific cytotoxic T lymphocyte (CTL) responses, effectively mediating the killing of OSCC cells, thereby highlighting its potential as an immunotherapeutic target for OSCC [12,13]. However, significant intratumoral heterogeneity of MAGED4 expression has been observed in tumors such as glioma and colon cancer [14,15]. This spatial heterogeneity may impede consistent antigen presentation, consequently affecting the efficacy of immunotherapy [16]. Currently, research on MAGED4 expression heterogeneity in OSCC remains limited; existing studies primarily rely on small sample cohorts and focus on overall expression levels, lacking in-depth exploration of heterogeneity.

Emerging evidence indicates that epigenetic modifications, including DNA methylation and histone acetylation, play crucial roles in the heterogeneous regulation of MAGE gene expression [17,18]. However, the epigenetic mechanisms governing MAGED4 expression in OSCC remain undefined. The presence of a CpG island within the *MAGED4* promoter region [9] implies a potential regulatory role for DNA methylation, although this has yet to be experimentally validated in OSCC. Furthermore, the involvement of histone acetylation in MAGED4 regulation, as well as the possible synergistic interaction between DNA methylation and histone acetylation in modulating MAGED4 expression, requires further investigation.

This study systematically investigates the expression and clinical significance of MAGED4 in OSCC, with a focus on its epigenetic regulation. Through integrating online databases, clinical samples, and single-cell RNA sequencing, we characterized the heterogeneous expression of MAGED4 in OSCC. Furthermore, we experimentally confirmed that MAGED4 transcription is suppressed by DNA methylation and potentially regulated by histone acetylation. Additionally, we evaluated the synergistic reactivation of MAGED4 using DNA methyltransferase inhibitors and histone deacetylase inhibitors in OSCC cell models, exploring the underlying mechanisms from an epigenetic perspective. These findings provide experimental insights into the regulatory mechanisms governing MAGED4 heterogeneity and establish a foundation for exploring epigenetic-based therapeutic strategies to improve immunotherapeutic outcomes.

## 2. Results

### 2.1. Elevated Expression of MAGED4 in OSCC

Previous studies have reported elevated MAGED4 expression in OSCC [10,11]; however, these investigations were primarily based on limited clinical samples. To achieve a more comprehensive evaluation, we analyzed *MAGED4* expression using large-scale public databases and our in-house clinical cohort.

**Figure 1:**
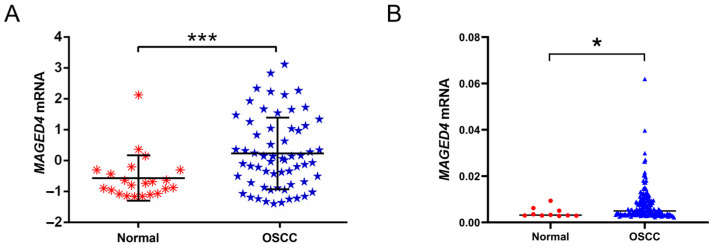
*MAGED4* mRNA is consistently upregulated in OSCC across independent transcriptomic cohorts. (**A**) *MAGED4* mRNA expression in 62 oral squamous cell carcinoma (OSCC) tissues and 22 normal oral mucosa from the Oncomine database. Scatter plots show individual sample values. *MAGED4* expression is significantly higher in OSCC tissues compared with normal controls. (**B**) Validation of *MAGED4* overexpression using 338 OSCC tissues and 32 paracancerous normal tissues from The Cancer Genome Atlas (TCGA) database. Each point represents transcript abundance for an individual sample. OSCC specimens show significantly increased *MAGED4* mRNA levels relative to normal tissues. *: *p* < 0.05; ***: *p* < 0.001.

Analysis of the Oncomine OSCC dataset (*n* = 62) revealed a significant increase of *MAGED4* mRNA expression in tumor tissues compared to normal controls (*p* < 0.001). Notably, heterogeneous expression was observed among OSCC samples (Figure 1A). These findings were further corroborated in the larger The Cancer Genome Atlas (TCGA) OSCC cohort (*n* = 338), which also demonstrated significant upregulation of *MAGED4* mRNA (*p* < 0.05) and exhibited similar heterogeneity (Figure 1B).

To validate these findings at the protein level, Immunohistochemistry (IHC) was performed on our clinical cohort comprising 120 OSCC tissues, 24 oral leukoplakia (OLK) tissues, and 29 adjacent normal oral tissues.

**Figure 2:**
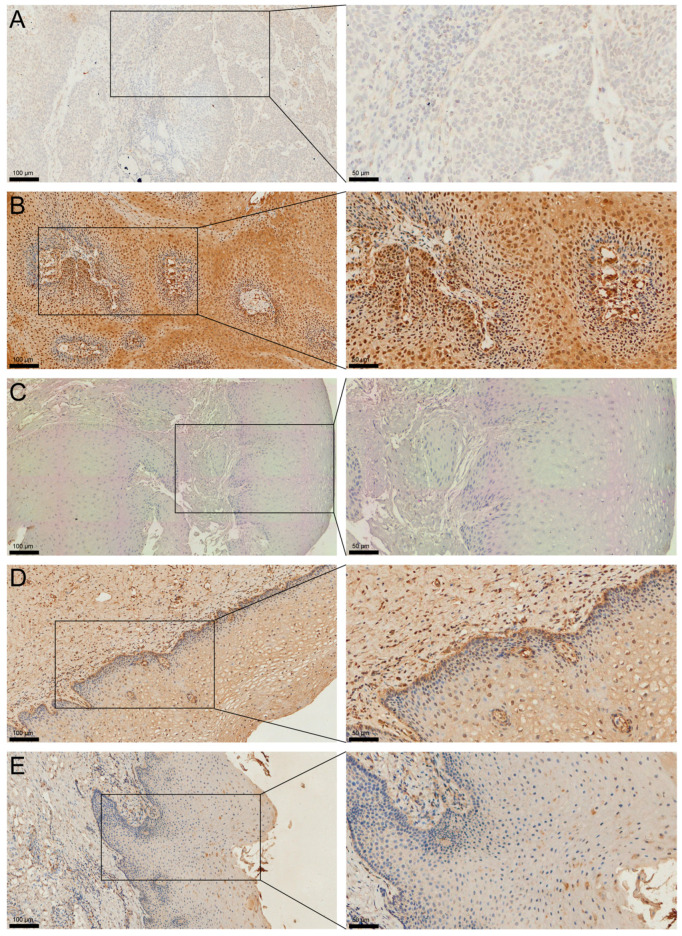
MAGED4 protein is highly expressed in OSCC and precancerous lesions. (**A**,**B**) Representative images of MAGED4 immunohistochemical staining in OSCC tissues showing low expression (**A**) and high expression (**B**). (**C**,**D**) Representative images of oral leukoplakia (OLK) tissues showing low expression (**C**) and high expression (**D**). (**E**) Representative image of adjacent normal oral mucosa showing consistently low MAGED4 expression. MAGED4 immunoreactivity was predominantly nuclear with variable cytoplasmic staining. Insets on the right show higher-magnification views of boxed areas. Scale bars: 100 μm (left panels) and 50 μm (right panels).

MAGED4 protein showed variable staining intensities, ranging from yellowish to brownish, with either homogeneous or granular distribution patterns. The subcellular localization of MAGED4 was predominantly nuclear, though distinct cytoplasmic immunoreactivity was also observed. In addition to its expression in tumor cells within OSCC tissues, MAGED4 is also detected in some of the endothelial cells and fibroblasts (Figure S2). High MAGED4 protein expression was detected in 71.7% (86/120) of OSCC samples, whereas 28.3% (34/120) showed low expression (Figure 2A,B). Among the OLK tissues, 54.2% (13/24) exhibited high MAGED4 expression, and 45.8% (11/24) showed low expression (Figure 2C,D). In contrast, all 29 adjacent normal oral tissues demonstrated low or undetectable MAGED4 protein expression (Figure 2E).

Taken together, these results indicate that MAGED4 is frequently overexpressed at both mRNA and protein levels in OSCC tissues, suggesting its potential role in oral carcinogenesis.

### 2.2. Heterogeneous Expression of MAGED4 in OSCC

**Figure 3:**
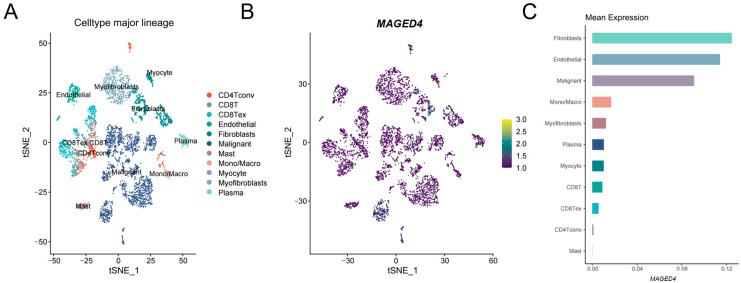
*MAGED4* exhibits heterogeneous expression within the OSCC tumor microenvironment at single-cell level. (**A**) t-Distributed stochastic neighbor embedding (t-SNE) plotting showing the major cell lineages identified from single-cell RNA sequencing of OSCC samples. Each cluster is color-coded and annotated according to its inferred cell type. (**B**) t-SNE feature plot displaying the expression distribution of *MAGED4* across all annotated cell clusters. Expression intensity is represented according to a color scale (purple = low expression; yellow = high expression), revealing heterogeneous and cell-type-dependent expression patterns. (**C**) Bar chart summarizing the mean *MAGED4* expression levels across each major cell lineage, highlighting the cell types with relatively higher transcriptional abundance.

After confirming MAGED4 overexpression in bulk OSCC tissues at both mRNA and protein levels, we next analyzed its expression patterns at the single-cell level resolution within the tumor microenvironment. Using a public single-cell RNA-sequencing dataset, we performed quality control, normalization, and clustering, identifying 11 distinct cell populations (Figure 3A). *MAGED4* overexpression was mainly found in three cell types: fibroblasts, endothelial cells, and malignant cells, each showing considerable heterogeneity in expression. In contrast, immune cells (such as T cells and macrophages) and normal epithelial cells exhibited minimal *MAGED4* expression (Figure 3B,C). This heterogeneous expression pattern suggests that uneven antigen presentation may limit the effectiveness of MAGED4-targeted immunotherapy.

### 2.3. Clinical Correlates of MAGED4 Expression in OSCC

**Figure 4:**
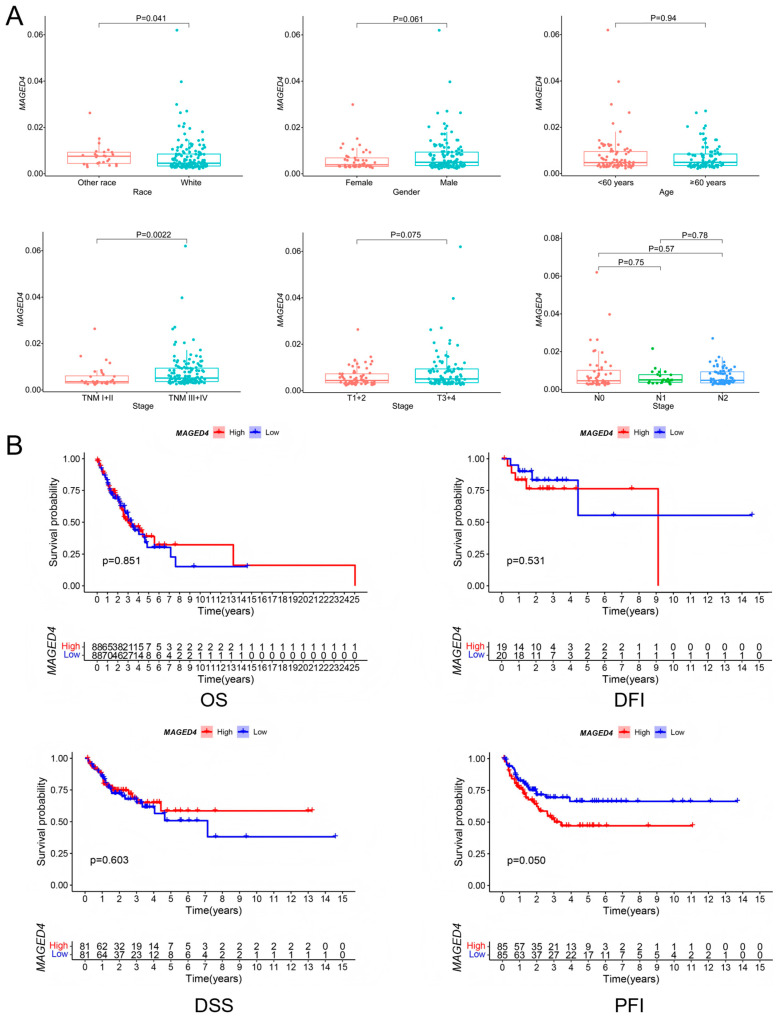
Associations between *MAGED4* mRNA expression and clinicopathological features as well as survival outcomes in the TCGA OSCC cohort. (**A**) Association analysis of *MAGED4* mRNA expression with clinicopathological parameters. Statistical significance was assessed using the indicated *p*-values. Differences were most notable across race and overall TNM stage, while other variables showed no significant associations. (**B**) Kaplan-Meier survival analyses evaluating the prognostic value of *MAGED4* expression in OSCC. Patients were stratified into high- and low-expression groups, and survival curves were generated for overall survival (OS), disease-free interval (DFI), disease-specific survival (DSS), and progression-free interval (PFI). Log-rank tests were used for statistical comparison. While no significant differences were observed for OS, DFI, or DSS, a borderline association was detected for PFI (*p* = 0.050).

Following the characterization of MAGED4 expression pattern in OSCC, we evaluate its clinical significance. OSCC patient samples from the TCGA database were divided into low- and high-*MAGED4* mRNA groups based on the median expression levels. Correlation analyses revealed that elevated *MAGED4* mRNA expression was significantly associated with advanced clinical stage (III–IV vs. I–II; *p* < 0.01) and ethnicity (lower in Caucasians vs. other races; *p* < 0.05), but not with gender, age, T stage, or N stage (Figure 4A). Survival analysis revealed that high *MAGED4* mRNA expression was not significantly associated with overall survival (OS), disease-free interval (DFI), or disease-specific survival (DSS). However, a borderline correlation was observed for progression-free interval (PFI) with a *p*-value of 0.05, suggesting a possible role of MAGED4 in tumor progression (Figure 4B).

IHC analysis further revealed no significant associations between MAGED4 protein expression and clinicopathological parameters, including gender, age, tumor size, differentiation, TNM stage, lymph node metastasis, and the expression of ki-67, p53 and p16 (Table 1).

### 2.4. Suppression of MAGED4 Transcriptional Activity by Promoter Methylation

Since DNA methylation is a key epigenetic mechanism and a CpG island is located in the *MAGED4* promoter [9,19], we investigated whether methylation of this region directly regulates *MAGED4* transcription.

**Figure 5:**
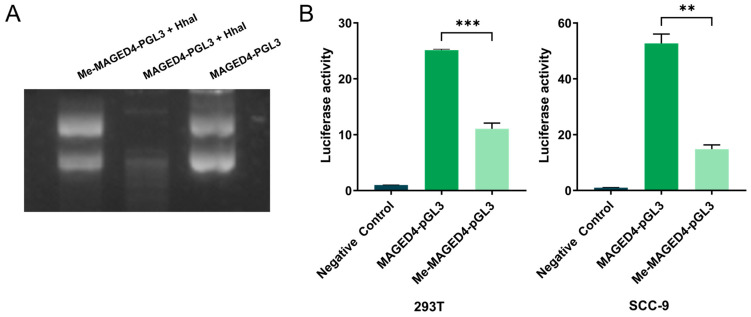
DNA methylation of the *MAGED4* promoter directly represses its transcriptional activity. (**A**) Validation of in vitro methylation of the *MAGED4* promoter using HhaI restriction enzyme digestion. Methylated reporter plasmid (Me-MAGED4-pGL3) remained undigested in the presence of HhaI, confirming protection from restriction due to methylation. In contrast, the unmethylated construct (MAGED4-pGL3 + HhaI) showed complete digestion, while the undigested MAGED4-pGL3 lane served as a control for the original plasmid size. (**B**) Dual-luciferase reporter assays evaluating the effect of promoter methylation on *MAGED4* transcriptional activity in 293T and SCC-9 cells. Cells transfected with the unmethylated MAGED4-pGL3 construct showed markedly higher luciferase activity compared with those transfected with the methylated Me-MAGED4-pGL3 construct, indicating repression of promoter activity by methylation. **: *p* < 0.01 and ***: *p* < 0.001.

To this end, we developed an in vitro methylation model of the *MAGED4* promoter and confirmed methylation status through HhaI restriction enzyme digestion. As shown in Figure 5A, the methylated reporter construct (Me-MAGED4-pGL3) remained resistant to HhaI digestion, indicating successful methylation. Conversely, the unmethylated control (MAGED4-pGL3) was fully cleaved, confirming the specificity and completeness of methylation.

Subsequently, we transfected these constructs into HEK293T and SCC-9 cells to evaluate the impact of promoter methylation on transcriptional activity. Luciferase reporter assay revealed that Me-MAGED4-pGL3 significantly repressed luciferase activity compared to the unmethylated MAGED4-pGL3 in both cell lines (*p* < 0.001, *p* < 0.01, respectively; Figure 5B). These findings demonstrate that methylation of the *MAGED4* promoter directly inhibits its transcriptional activity, supporting a regulatory role for promoter methylation in *MAGED4* gene expression.

### 2.5. Implication of MAGED4′s Involvement in Histone Acetylation via Co-Expression Network

**Figure 6:**
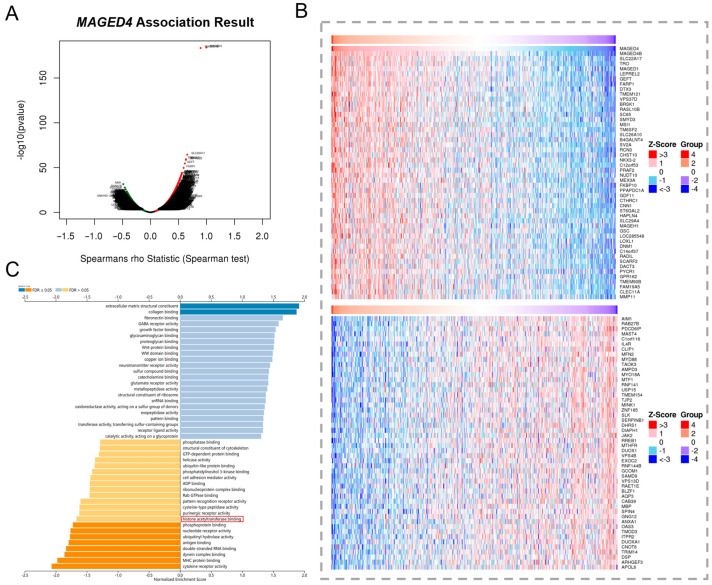
Co-expression landscape of *MAGED4* in OSCC reveals enrichment of histone acetylation-related pathways. (**A**) Volcano Plot showing genes correlated with *MAGED4* expression from the TCGA-OSCC cohort. Genes with significant positive correlations are highlighted in red, whereas those with significant negative correlations are highlighted in green. (**B**) Heatmap showing the top 50 genes most strongly associated with *MAGED4* expression. Upper panel: top positively correlated genes; lower panel: top negatively correlated genes. Expression values are displayed as Z-scores, with red indicating higher expression and blue indicating lower expression across OSCC samples. (**C**) Gene Ontology (GO) enrichment analysis of *MAGED4*-associated genes, ordered by normalized enrichment score (NES). Red box indicate the correlation between *MAGED4* expression and histone acetyltransferase binding.

To explore other regulatory mechanisms underlying the heterogeneity in MAGED4 expression, we performed a co-expression analysis using the TCGA database (Figure 6A). The analysis identified the top 50 genes most strongly correlated with *MAGED4* expression (Figure 6B). Gene Ontology (GO) enrichment analysis of these genes revealed a significant enrichment of epigenetic binding activities among these co-expressed genes, particularly histone acetyltransferase binding (Figure 6C). Suggesting that histone acetylation may also contribute to MAGED4 regulation.

### 2.6. Attenuation of MAGED4 Expression Heterogeneity in OSCC by Epigenetic Drugs

Based on the epigenetic basis for heterogeneous MAGED4 expression, we hypothesized that a combined epigenetic targeting could homogenize its expression. To evaluate this, two OSCC cell lines, CAL-27 and SCC-9, were treated with the epigenetic drugs 5-aza-2′-deoxycytidine (DAC), trichostatin A (TSA), and valproic acid (VPA).

**Figure 7:**
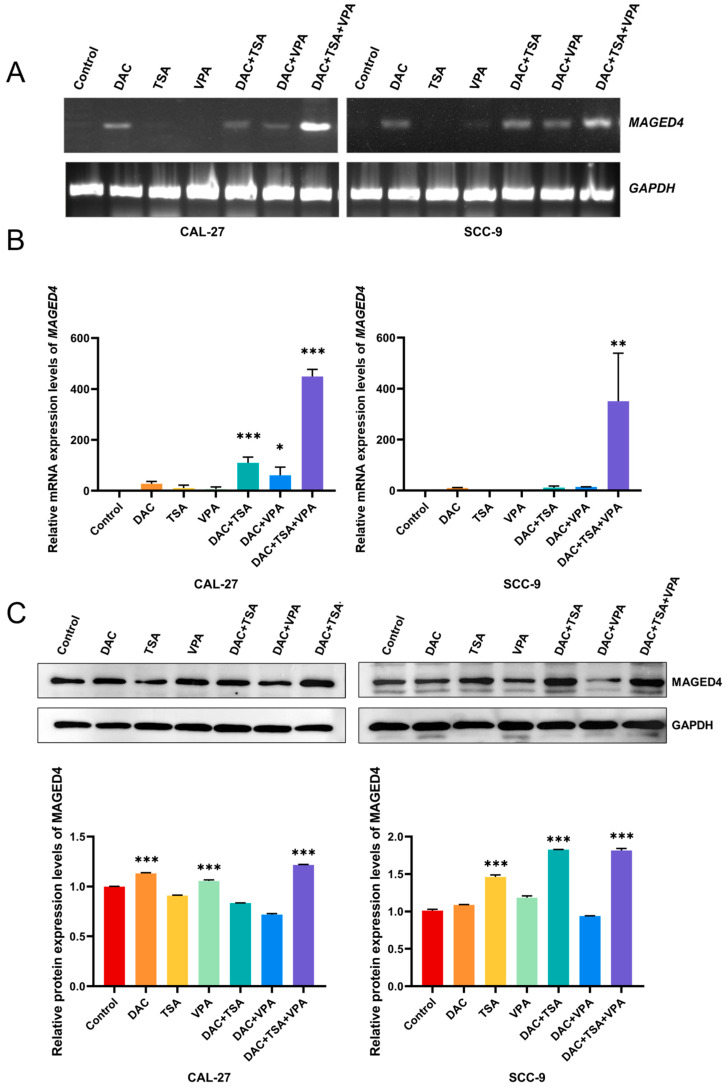
Epigenetic drug treatment induces MAGED4 expression in OSCC cell lines. (**A**) Reverse transcription polymerase chain reaction (RT-PCR) analysis of *MAGED4* mRNA expression in CAL-27 and SCC-9 cells following treatment with individual epigenetic agents—5-Aza-2′-deoxycytidine (DAC), Trichostatin A (TSA), or Valproic acid (VPA)—and their combinations (DAC + TSA, DAC + VPA, DAC + TSA + VPA). GAPDH served as a loading control. (**B**) Quantitative RT-PCR (qRT-PCR) quantification of *MAGED4* mRNA expression in CAL-27 and SCC-9 cells after the indicated treatments. All values are normalized to GAPDH and expressed relative to untreated controls. Combined DAC + TSA + VPA treatment produced the strongest induction of *MAGED4* transcript levels in both cell lines. (**C**) Western blot analysis and quantification of MAGED4 protein expression after the same epigenetic treatments in CAL-27 and SCC-9 cells. Representative blot images are shown, with GAPDH as a loading control. Quantified protein levels demonstrate that combination treatments markedly elevate MAGED4 protein abundance. *: *p* < 0.05; **: *p* < 0.01; ***: *p* < 0.001 vs. control.

Reverse transcription polymerase chain reaction (RT-PCR) analysis showed that TSA or VPA alone did not induce *MAGED4* mRNA expression in either cell line, while DAC alone and its combinations with TSA or VPA upregulated *MAGED4* transcripts. The triple combination (DAC, TSA, and VPA) produced the strongest induction (Figure 7A). To quantitatively confirm these findings, quantitative RT-PCR (qRT-PCR) was performed. The triple-drug combination induced a 449-fold increase in *MAGED4* mRNA in CAL-27 (*p* < 0.001), significantly exceeding the effects of dual combinations (DAC + TSA: 109-fold, *p* < 0.001; DAC + VPA: 60-fold, *p* < 0.05). In SCC-9 cells, the triple-drug treatment led to a 350.4-fold increase (*p* < 0.01), whereas the dual combinations showed non-significant induction (11.1-fold for DAC + TSA, and 14.2-fold for DAC + VPA. Notably, no single-agent treatments significantly induce *MAGED4* mRNA expression in either cell line (*p* > 0.05) (Figure 7B).

To determine whether mRNA changes resulted in protein expression, Western blot analysis was performed. In CAL-27 cells, the triple-drug combination induced a 1.2-fold protein increase in MAGED4 protein (*p* < 0.001), consistent with its robust mRNA activation. Notably, DAC and VPA alone increased protein despite minimal mRNA changes, whereas TSA alone and the dual combination had no significant effects. Similarly, in SCC-9 cells, significant protein induction was observed with the triple-drug treatment (1.8-fold increase, *p* < 0.001), as well as with DAC + TSA (1.8-fold, *p* < 0.001) and TSA alone (1.4-fold, *p* < 0.001). No significant protein changes were detected following DAC + VPA, DAC alone or VPA alone (Figure 7C).

Collectively, these findings demonstrate that triple-drug epigenetic therapy effectively activates MAGED4 expression, highlighting the importance of combinatorial epigenetic modulation and suggesting that personalized epigenetic strategies may enhance OSCC therapy.

### 2.7. Regulation of MAGED4 Promoter Methylation by Epigenetic Drugs

**Figure 8:**
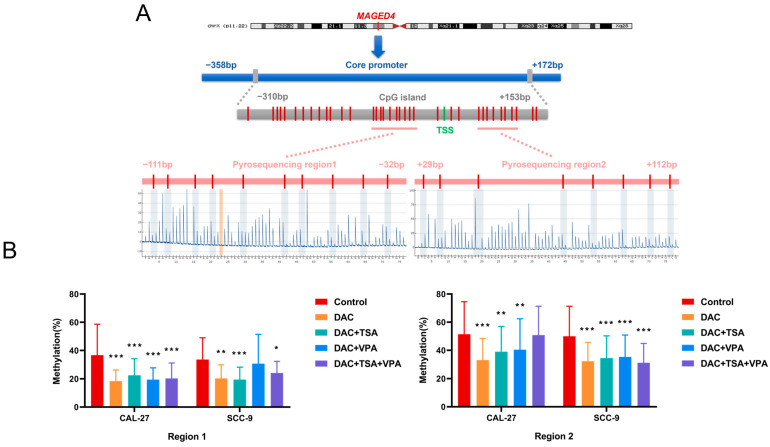
Epigenetic drug treatment reduces DNA methylation of the *MAGED4* promoter in OSCC cell lines. (**A**) Schematic illustration of the *MAGED4* promoter, including the CpG island, core promoter region, transcription start site (TSS), and the two pyrosequencing target regions (Region 1 and Region 2). The positions of individual CpG sites analyzed by pyrosequencing are indicated by red vertical bars. Approximate distances upstream and downstream of the TSS are shown to provide genomic context. (**B**) Quantitative analysis of DNA methylation levels in CAL-27 and SCC-9 cells following treatment with DAC alone or in combination with TSA and/or VPA. Methylation percentages for each treatment group are presented as mean ± SD for Region 1 (left panel) and Region 2 (right panel). Data are presented as mean ± SD. Significant differences relative to the control are indicated: *: *p* < 0.05, **: *p* < 0.01, ***: *p* < 0.001.

To determine whether epigenetic drug-induced MAGED4 upregulation involves DNA methylation, we focused on the CpG island in the core promoter region of *MAGED4* [20]. *MAGED4* methylation status was quantitatively assessed via pyrosequencing 18 CpG sites, which were grouped into two regions (region 1 and region 2). The schematic representation of the CpG island and the analyzed regions is shown in Figure 8A.

Given that DAC is a well-established inhibitor of DNA methyltransferase (DNMT), we examined the effects of DAC, alone or in combination with TSA/VPA, on *MAGED4* promoter methylation. As illustrated in Figure 8B, in region 1 of CAL-27 cells, all treatment groups (DAC alone, DAC + TSA, DAC + VPA, and DAC + TSA + VPA) exhibited a remarkable reduction in methylation compared to the controls (*p* < 0.001). In SCC-9 cells, significant hypomethylation in region 1 was observed following treatment with DAC alone, DAC + TSA, and DAC + TSA + VPA (*p* < 0.05), whereas the DAC + VPA group did not exhibit a statistically significant change. In region 2 of CAL-27 cells, significant hypomethylation was induced by DAC alone and both dual-combination treatments (DAC + TSA and DAC + VPA) (*p* < 0.01), while the triple-drug combination did not result in significant demethylation. Conversely, in SCC-9 cells, all treatments resulted in pronounced hypomethylation (*p* < 0.001), with the triple-drug combination demonstrating the strongest effect.

Overall, most treatments significantly reduced the methylation of the *MAGED4* promoter, except the DAC + VPA combination in SCC-9 (region 1) and the triple-drug combination in CAL-27 (region 2).

### 2.8. Modulation of Histone H3 Acetylation at the MAGED4 Promoter by Epigenetic Drugs

To determine whether the upregulation of MAGED4 expression by epigenetic drugs involves histone acetylation at its promoter, we investigated the histone acetylation levels in CAL-27 and SCC-9. Preliminary experiments revealed that CAL-27 cells did not exhibit significant changes in histone acetylation enrichment at the *MAGED4* promoter following treatment (Figure S1). Consequently, subsequent functional experiments focused on SCC-9 cells.

**Figure 9:**
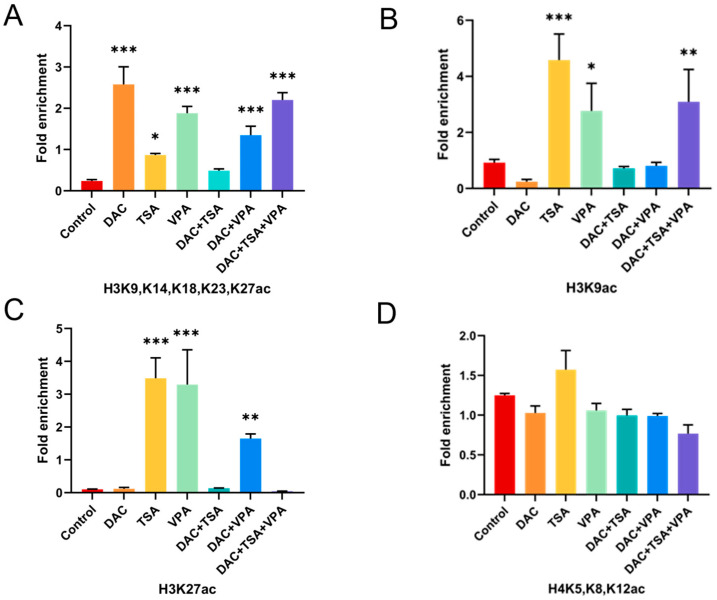
Epigenetic drug treatments enhance histone acetylation at the *MAGED4* promoter in OSCC cells. (**A**–**D**) Chromatin immunoprecipitation followed by qPCR (ChIP-qPCR) was performed to assess histone acetylation levels at the *MAGED4* promoter after treatment with DAC, TSA, VPA, or their combinations. Enrichment values are shown as fold change relative to the untreated control. (**A**) Enrichment of pan-acetylated histone H3 (H3ac; acetylation at K9, K14, K18, K23, and K27). (**B**) Enrichment of H3K9ac. (**C**) Enrichment of H3K27ac. (**D**) Enrichment of pan-acetylated histone H4 (H4ac; acetylation at K5, K8, and K12). Data are presented as mean ± SD; *: *p* < 0.05, **: *p* < 0.01, ***: *p* < 0.001 vs. control.

Chromatin immunoprecipitation-qPCR (ChIP-qPCR) was performed using a pan-acetylated histone H3 (recognizing K9, K14, K18, K23, K27). We found that most drug treatments significantly increased H3 acetylation at the *MAGED4* promoter, except for the combination of DAC and TSA (Figure 9A).

Given the established roles of H3K9ac and H3K27ac in transcriptional activation [21,22,23], we performed targeted ChIP-qPCR using antibodies specific for H3K9ac and H3K27ac. Treatment with TSA, VPA alone, and the three-drug combination significantly elevated H3K9 acetylation levels compared to controls (Figure 9B). Similarly, H3K27ac enrichment was observed following treatment with TSA, VPA, and the DAC + TSA combination (Figure 9C). In contrast, no significant enrichment of acetylated histone H4 was detected at the *MAGED4* promoter across all treatment groups (Figure 9D).

These findings suggest that epigenetic drugs modulate MAGED4 expression, at least in part, through site-specific acetylation of histone H3 (particularly K9 and K27), while histone H4 acetylation does not appear to be involved in this modulation mechanism.

## 3. Discussion

Tumor heterogeneity, particularly the variable expression of tumor-associated antigens such as MAGE family members, remains a significant challenge to effective immunotherapy [18,24,25,26]. In OSCC, MAGE antigens exhibit substantial variability in expression. Despite this recognition, the regulatory mechanisms driving this heterogeneity are still poorly characterized. This hampers the development of targeted interventions aimed at normalizing antigen expression and improving immunotherapeutic outcomes. Given the well-established role of epigenetic modifications such as DNA methylation and histone acetylation in mediating gene expression heterogeneity in tumors [27,28], precisely identifying key MAGE antigens, elucidating the epigenetic mechanisms governing their expression heterogeneity, and developing strategies to enhance immunotherapeutic responses are of significant translational value.

In this study, we showed that the mRNA and protein levels of MAGED4 were significantly higher in OSCC tissues than adjacent normal tissues. These findings were validated through analyses of online databases and clinical specimens, derived from a larger patient cohort. Our results corroborate earlier reports by Cheong [11] and Chong et al. [10], which observed similar trends but utilized smaller sample sizes. Notably, our analysis included OLK, a well-recognized precancerous lesion with well-documented malignant potential [29]. High MAGED4 expression was detected in 71.7% (86/120) of OSCC tissues and 54.2% (13/24) of OLK tissues, whereas it was absent or minimally expressed in all normal oral mucosal tissues. To our knowledge, this is the first report to demonstrate early MAGED4 upregulation in oral precancerous lesions. These findings suggest that MAGED4 expression may be upregulated during the initial stages of oral mucosal malignant transformation, highlighting its potential as a biomarker for early screening and risk stratification in oral cancer. Moreover, as an emerging cancer therapy, PD-L1-targeted therapy has spawned various combination regimens, which are more effective than monotherapy [4], similar to MAGED4, PD-L1 exhibits a high expression profile in oral squamous cell carcinoma (OSCC) [30,31], providing a rational basis for the development of dual-targeted therapeutic strategies co-targeting MAGED4 and PD-L1, thereby offering a novel and effective treatment option for OSCC patients.

At the cellular level, IHC analysis revealed that MAGED4 protein was predominantly localized within the nucleus of OSCC cells, with concurrent expression observed in the cytoplasm. This finding contrasts with the results reported by Chong et al. [10], who reported predominantly cytoplasmic MAGED4 localization with some membrane co-expression. Both studies used antibodies from Sigma Aldrich, suggesting that the observed differences may stem from tissue heterogeneity—such as variations in genetic background, differentiation status, and tumor microenvironment among patient cohorts.

Such heterogeneity can influence the subcellular distribution of proteins, potentially accounting for differences in localization patterns. Despite these differences, both studies confirm that MAGED4 exhibits a dynamic distribution in OSCC, including localization within the nucleus, cytoplasm, and membrane. This likely reflects its functional complexity during tumor progression; for instance, nuclear localization may be associated with transcriptional regulation, while cytoplasmic or membrane localization could be involved in signal transduction or protein interactions, though this requires further validation.

Beyond cellular localization, further analysis demonstrated that *MAGED4* expression in OSCC correlates with clinical stage and ethnicity. Specifically, the association with clinical stage suggests its potential role in regulating malignant progression, while ethnic differences may be attributed to population-specific factors such as genetic background and environmental factors, which require further validation. In prognosis analysis, *MAGED4* mRNA levels showed no significant association with OS, DFI, or DSS. However, a marginal association with PFI was observed, implying a potential role in early tumor progression. This finding contrasts with the study by Chong et al. [10], which reported MAGED4 protein expression correlated with poor DFS. Three factors may explain this discrepancy: first, mRNA levels do not always correspond to protein abundance or function; second, differences in cohort source, size, and tumor heterogeneity; and third, varied prognostic endpoints (our study’s focus on PFI versus their focus on DFS).

MAGED4’s expression profile in OSCC supports its potential as an immunotherapy target. Lim et al. [12] identified multiple human leukocyte antigen (HLA)-restricted immunogenic peptides derived from MAGED4B, demonstrating their capability to induce effective cytotoxic T lymphocyte (CTL) responses and cytotoxic effects in vitro. Chai et al. [13] further developed a bivalent peptide vaccine (PV1) containing MAGED4B and FJX1 antigens. This vaccine exhibited enhanced immunogenicity and synergistic anti-tumor effects in head and neck cancer patients, providing strong experimental evidence for targeting MAGED4 in OSCC immunotherapy. However, both single-cell transcriptome and IHC analyses revealed significant MAGED4 expression heterogeneity in OSCC tissues: high expression in some cell populations and negativity in others. This pattern aligns with reports in gliomas, colon cancer, and other tumor types [14,15]. Such differential antigen distribution may cause a “partial antigen exposure and partial immune escape” phenomenon: tumor cells with low/negative MAGED4 expression evade immune recognition, while only MAGED4-positive subpopulations are targeted. This incomplete targeting likely reduces the efficacy of immunotherapy and may promote treatment resistance or recurrence. Therefore, achieving homogeneous MAGED4 expression across OSCC cell populations is critical for improving immunotherapy outcomes.

Epigenetic modifications, such as DNA methylation and histone acetylation, play a crucial role in regulating the heterogeneity observed in tumor gene expression [32,33]. Since the *MAGED4* promoter contains a CpG island [20], we confirmed, via in vitro promoter methylation and luciferase reporter assays, that promoter methylation suppresses MAGED4 transcriptional activity, consistent with our prior findings in glioma [9,20]. Additionally, a functional analysis of genes co-expressed with MAGED4 demonstrated significant associations with histone acetyltransferases and chromatin remodelers, suggesting that histone acetylation may also play a role in regulating MAGED4 expression. Given the known synergy between DNA methylation and histone deacetylation in gene silencing [33,34,35], we designed different drug regimens, including single agents, dual combinations, and triple combinations, using DAC (a DNA methyltransferase inhibitor) and TSA/VPA (histone deacetylase inhibitors). Although single-cell sequencing shows heterogeneous *MAGED4* expression across different cell types in tumors, we used OSCC cell lines to evaluate drug-induced MAGED4 expression, as tumor cells are the most abundant component of tumor tissue and the primary target of anti-tumor immunity.

Subsequently, drug induction experiments revealed that the triple-drug combination (DAC, TSA, VPA) exerted the most potent synergistic effect in inducing MAGED4 expression—particularly at the mRNA level, with a several-hundred-fold increase. However, the patterns of mRNA and protein induction were inconsistent. In CAL-27 cells, DAC or VPA monotherapy, and in SCC-9 cells, TSA and DAC + TSA combinations increased protein levels without significant mRNA changes, implying post-transcriptional or translational regulation. Mechanistically, the efficient reactivation of MAGED4 was associated with coordinated remodeling of DNA methylation and histone acetylation. Specifically, most regimens with DAC induced significant demethylation at two key regions of the *MAGED4* promoter, and though the triple combination’s demethylation effect in CAL-27’s region 2 was not statistically significant, its strong transcriptional activation suggests possible compensatory mechanisms; For histone acetylation, preliminary experiments revealed no significant enrichment in treated CAL-27 cells; accordingly, subsequent studies were focused on SCC-9 cells, which exhibit greater sensitivity to histone modification changes. This difference in cellular response is likely due to the distinct genetic backgrounds of the cell lines. In SCC-9 cells, drug-regulated *MAGED4* expression closely correlated with specific histone H3 acetylation, as TSA, VPA monotherapies, and the triple combination all significantly enhanced the enrichment of *MAGED4* promoter by H3K9ac and H3K27ac. Taken together, these results support a synergistic regulation model: DAC-mediated DNA demethylation may remove methyl-binding proteins such as MeCP2 that recruit histone deacetylase (HDAC) complexes, creating a more open chromatin state; concurrently, TSA and VPA inhibit HDACs, directly promoting histone H3 acetylation, particularly at K9 and K27. Together, these two mechanisms synergistically reverse MAGED4’s epigenetic silencing and significantly upregulate its transcription [36,37].

Despite these findings, the study has limitations. Single-cell sequencing analysis was performed using the HNSC dataset, which may introduce potential bias. Additionally, partial missingness of clinical metadata could potentially compromise the accuracy of correlation analyses between *MAGED4* and clinical parameters, as well as survival analyses. Then, the clinical sample size was small, and in vivo models are lacking. Finally, the specific methyl-CpG binding domain (MBD)-HDAC complexes repressing MAGED4 remain unidentified. To address these limitations, future studies could validate its prognostic value by expanding sample sizes, establishing in vivo models to evaluate the efficacy of combined regimens, and characterizing the specific composition of MBD-HDAC complexes. Building on these efforts, combining epigenetic drugs with MAGED4-targeted immunotherapy is expected to overcome heterogeneity and enhance anti-tumor activity.

## 4. Materials and Methods

### 4.1. Integrated Bioinformatic Analysis

The mRNA expression of *MAGED4* was initially investigated using an OSCC dataset available on the Oncomine platform (https://www.oncomine.org/), which included 62 tumor tissues and 22 normal oral mucosa samples. This preliminary analysis was subsequently validated using the Head and Neck Squamous Cell Carcinoma (HNSC) cohort from TCGA (https://portal.gdc.cancer.gov/), with a specific focus on the OSCC subset comprising 338 tumor samples and 32 paracancerous tissue samples.

For subsequent analysis, the TCGA-OSCC cohort was stratified into high- and low-expression groups based on the median level of *MAGED4* mRNA expression. The association of *MAGED4* mRNA expression with clinicopathological parameters and patient survival was then evaluated. Furthermore, Genes co-expressed with *MAGED4* were identified within the OSCC cohort using the LinkedOmics database (https://linkedomics.org). GO enrichment analysis was performed on the top 50 co-expressed genes to infer potential biological functions.

### 4.2. Single-Cell RNA Sequencing (scRNA-Seq) Data Processing

The scRNA-seq dataset GSE103322 (H5 format) and its cell annotation file were retrieved from the TISCH database (http://tisch.comp-genomics.org). Data analysis was conducted using the R packages MAESTRO (version 1.8.0) and Seurat (version 5.1.0) [38]. Briefly, raw data underwent stringent quality control to remove low-quality cells and genes. This was followed by data normalization, identification of highly variable genes, and principal component analysis (PCA). Key principal components were selected for further analysis based on statistical significance criteria. Subsequently, cells were clustered using t-distributed stochastic neighbor embedding (t-SNE). Cell types were annotated based on established lineage markers and reference annotations from TISCH. The expression distribution of *MAGED4* was depicted on the t-SNE plot, and its average expression levels across different cell types were compared using a bar chart.

### 4.3. Tissue Samples

Formalin-fixed, paraffin-embedded tissue sections from 120 OSCC, 24 OLK, and 29 normal oral mucosal samples were acquired from the Department of Pathology at the People’s Hospital of Guangxi Zhuang Autonomous Region between January 2018 and January 2021. The normal control samples were derived from adjacent non-cancerous tissues of OSCC patients. The OSCC cohort consisted of 89 males and 31 females (median age, 58 years; range, 28–85 years) and was stratified into two groups based on the median age. Clinicopathological characteristics of the patients are summarized in Table 1. All tumor diagnoses were independently confirmed by two experienced pathologists. Informed consent was obtained from each patient. The study was conducted in accordance with the Declaration of Helsinki, and the protocol was approved by the Ethics Committee of the People’s Hospital of Guangxi Zhuang Autonomous Region (Approval No. 2018-18) on 17 May 2018.

### 4.4. IHC

IHC staining was performed using the PV-6000 Mouse/Rabbit Polymer Detection System (ZSGB-BIO, Beijing, China) according to the manufacturer’s instructions. Briefly, after dewaxing and hydration, antigen retrieval was conducted using sodium citrate buffer (pH 6.0) in a pressure cooker for 10 min. The sections were then blocked and incubated overnight at 4 °C with anti-MAGED4 antibody (1: 500, HPA003554, Sigma Aldrich, St. Louis, MO, USA). Following incubation with the secondary antibody, the immunoreactivity was visualized using 3,3′-diaminobenzidine (DAB), and nuclei were counterstained with hematoxylin.

IHC results were scored based on staining intensity and the percentage of positive tumor cells [14]. The percentage of positive cells was scored from 0 to 4 (0: ≤5%; 1: 6–25%; 2: 26–50%; 3: 51–75%; 4: >75%). The staining intensity was graded as 0 (negative), 1 (light yellow), 2 (brownish yellow), or 3 (tan). The final score (0–7) was obtained by summing these two scores and was categorized as follows: negative (−) for 0–1, weakly positive (+) for 2–3, moderately positive (++) for 4–5, and strongly positive (+++) for 6–7. For statistical analysis, the “−” and “+” groups were defined as low expression, while “++” and “+++” were defined as high expression.

### 4.5. Cell Culture and Pharmacological Interventions

OSCC cell lines (CAL-27 and SCC-9) and human embryonic kidney cell line (HEK293T) were obtained from the Cell Bank of Chinese Academy of Sciences (Shanghai, China). The CAL-27 and HEK293T cells were cultured in Dulbecco’s Modified Eagle Medium (DMEM), while the SCC-9 cells were maintained in DMEM/F12 medium. Both media were supplemented with 10% fetal bovine serum (FBS) and incubated at 37 °C in a humidified atmosphere containing 5% CO_2_.

DAC (A3656), VPA (99-66-1), and TSA (58880-19-6) were purchased from Sigma Aldrich (St. Louis, MO, USA). Cells in the logarithmic growth phase were seeded into 6-well plates and allowed to adhere for 24 h before drug exposure. Subsequently, the cells underwent the following treatments for a duration of five days: control (culture medium only); 1 μM DAC; 1 mM VPA; 1 μM TSA administered during the final 24 h; a combination of 1 μM DAC with 1 μM TSA added in the final 24 h; a combination of 1 μM DAC and 1 mM VPA; or a triple combination of 1 μM DAC and 1 mM VPA with 1 μM TSA added in the final 24 h. Treatments were renewed every 24 h, and all experimental procedures were performed under light-protected conditions.

### 4.6. Methylation Promoter Luciferase Reporter Assays

The core promoter fragment of *MAGED4* (−358 bp to +172 bp), as previously identified [20], was cloned into the KpnI and HindIII restriction sites of the pGL3-basic vector to generate the MAGED4-pGL3 plasmid. This recombinant plasmid was synthesized by Suzhou GenePharma (Suzhou, China). Subsequently, the MAGED4-pGL3 plasmid was subjected to in vitro methylation via incubation with CpG methylase (M.SssI, New England Biolabs, Ipswich, MA, USA) in NEB 2 Buffer, supplemented with 160 μM S-adenosylmethionine (SAM) at 37 °C for 2 h. Following the initial incubation, an additional 160 μM of fresh SAM was added, and the reaction was extended for another 2 h. The methylated plasmid (Me-MAGED4-pGL3) was then purified using the FastPure Gel DNA Extraction Mini Kit (Vazyme, Nanjing, China). Complete methylation was verified by assessing resistance to cleavage by the methylation-sensitive restriction enzyme HhaI (New England Biolabs, Ipswich, MA, USA).

Luciferase activity was measured using the Dual-Luciferase^®^ Reporter Assay System (E1910, Promega, Madison, WI, USA) according to the manufacturer’s protocol. Briefly, MAGED4-pGL3 or Me-MAGED4-pGL3 (expressing firefly luciferase) were co-transfected with the internal control vector pRLTK (expressing Renilla luciferase) into HEK293T and SCC-9 cells. After 48 h, the cells were lysed for 15 min at room temperature (RT). The firefly luciferase substrate was then introduced to the lysate, and the resulting firefly luciferase activity was measured in terms of Relative Light Units 1 (RLU1). Subsequently, the Renilla luciferase substrate was added, and its activity was recorded as Relative Light Units 2 (RLU2). The empty pGL3-basic vector served as a control to establish the basal luciferase activity. The relative luciferase activity, representing the promoter activity of *MAGED4*, was calculated as the ratio of RLU1 to RLU2.

### 4.7. RT-PCR and qRT-PCR

Total RNA was extracted from OSCC cell lines and reverse-transcribed into cDNA using the HiScript^®^ II 1st Strand cDNA Synthesis Kit (Vazyme, Nanjing, China). The expression of *MAGED4* was assessed by conventional RT-PCR and qRT-PCR as previously described [9]. For conventional RT-PCR, amplification was performed with *MAGED4*-specific primers (forward: 5′-CAGGATGGGAGGCAAGAGGACC-3′; reverse: 5′-CCAAGGAGGCGAGCTGAGGAGT-3′), and the products were visualized by electrophoresis on a 1.5% agarose gel. For qRT-PCR, a distinct set of *MAGED4* primers (forward: 5′-CCAGAATCAGAACCGAGA-3′; reverse: 5′-CCAAAATCTCCGTCCTCA-3′) was used with the ChamQ Universal SYBR qPCR Master Mix (Vazyme, Nanjing, China) on an ABI StepOne Plus system (Thermo Fisher Scientific, Waltham, MA, USA). In both assays, GAPDH served as the internal control, and relative mRNA expression levels were calculated using the 2^−ΔΔCt^ method.

### 4.8. Western Blot Analysis

OSCC cells were lysed in RIPA lysis buffer with protease inhibitors (Solarbio, Beijing, China). The supernatant was collected after centrifugation and denatured with loading buffer at 100 °C. Protein samples were then separated by SDS-PAGE and transferred to PVDF membranes. Subsequently, the membranes were blocked with 5% skimmed milk for 1 h, followed by an overnight incubation at 4 °C with primary antibodies against MAGED4 (1: 100, sc-398908, Santa Cruz Biotechnology, Santa Cruz, CA, USA) or GAPDH (1:1000, ab181602, Abcam, Cambridge, UK). After washing, the membranes were incubated with an HRP-conjugated secondary antibody (1:5000, Abcam, Cambridge, UK) for 2 h at RT. Protein bands were visualized using an FDbio-Pico ECL Kit (FD8000, Fdbio Science, Hangzhou, China) and captured with the Protein Simple FC3 system (Protein Simple, San Jose, CA, USA). Relative protein expression levels were determined by Image J software (version 1.54, NIH, Bethesda, Rockville, MD, USA).

### 4.9. Pyrosequencing Methylation Analysis

Genomic DNA was isolated from the OSCC cells using a DNA extraction kit (QIAGEN, Hilden, Germany) and then converted by Sodium Bisulfite using the EpiTect Bisulfite Kits (Qiagen, Hilden, Germany). Two regions of the *MAGED4* core promoter, comprising a total of 18 CpG sites, were PCR-amplified and sequenced on a PyroMark Q96 ID system using specific primers as described previously [15]. The primer sequences for each region were as follows: Region 1 (9 CpG sites) with 5′-TTGGAGGAAAGGGTTTTTGTTG-3′ (forward), 5′-CCCCATCCTATCTAAACTAAATCCTTAC-3′ (reverse), and 5′-GGGTTTTTGTTGGGAAA-3′ (sequencing); Region 2 (9 CpG sites) with 5′-GGTTGAGGGGTTTTTGGTGT-3′ (forward), 5’-AAAAACTCCTATCTAAACCTTAAATC-3′ (reverse), and 5′-GGTTTTTGGTGTTGAGGA-3’ (sequencing).

### 4.10. ChIP-qPCR

ChIP-qPCR was performed using the Pierce™ Agarose ChIP Kit (Thermo Fisher Scientific, Waltham, MA, USA) according to the manufacturer’s instructions. Briefly, OSCC Cells were cross-linked with 1% formaldehyde for 10 min and the reaction was quenched with 0.125 M glycine. The chromatin was subsequently fragmented using micrococcal nuclease and incubated with anti-histone H3/H4 acetylation antibody (PTM BIO, Hangzhou, China) at 4 °C overnight. The antibody-chromatin complexes were immunoprecipitated using Protein A/G agarose beads (Thermo Fisher Scientific, Waltham, MA, USA) for 1 h at 4  °C. The complexes were then reverse cross-linked at 65 °C for 40 min, and DNA was recovered after Proteinase K treatment by FastPure^®^ Cell/Tissue DNA Isolation Mini Kit (DC102, Vazyme, Nanjing, China). *MAGED4* promoter sequence in the resulting DNA fragments was amplified by qPCR. The primers used for the *MAGED4* promoter sequence were provided as follows: 5′-TTAACGTCATGGCACCACC-3′ (forward), 5′-AAACTCTATCCGGGCAAGAA-3′ (reverse). The calculation was carried out according to the Formula: Fold Enrichment = 2 − (CT(IP) − CT (Negative Control)).

### 4.11. Statistical Analysis

The data were presented as the means ± standard deviation from at least three independent experiments. Statistical analyses were performed using SPSS 26.0 (SPSS Inc., Chicago, IL, USA) and GraphPad Prism 8 (GraphPad Software, San Diego, CA, USA). For comparisons between two groups of continuous variables, the unpaired two-tailed Student’s *t*-test was used if the data followed a normal distribution; otherwise, the Mann-Whitney U test was applied. The Chi-square test or Fisher’s exact test was used to assess associations between MAGED4 expression and categorical clinicopathological parameters. Survival analysis was performed using the Kaplan-Meier method, and differences between groups were compared with the log-rank test. For comparisons among multiple groups, one-way or two-way ANOVA was used, followed by appropriate post-hoc tests. A *p*-value < 0.05 was considered statistically significant.

## 5. Conclusions

In conclusion, this study confirms that epigenetic drugs can overcome gene expression heterogeneity in OSCC. It provides critical experimental evidence and mechanistic insights for developing personalized MAGED4-based immunotherapy strategies in OSCC, laying a foundation for future translational research.

## Figures and Tables

**Table 1:** Relationship between MAGED4 protein expression and clinicopathological features of OSCC patients.

Clinicopathological Features	MAGED4 Protein		*χ* ^2^	*p*
	High Expression	Low Expression		
Genders				
Male	68(79.1)	21(61.8)	3.808	0.051
Female	18(20.9)	13(38.2)		
Age (years)				
≤58	46(53.5)	14(41.2)	1.477	0.224
>58	40(46.5)	20(58.8)		
Differentiation degree ^△^				
low-undifferentiated	15(17.6)	8(25.8)	0.951	0.329
high-moderately differentiated	70(82.4)	23(74.2)		
Tumor sizes (cm) ^△^ *				
≤2	24(38.7)	5(19.2)	3.146	0.076
>2	38(61.3)	21(80.8)		
Expression of ki-67 (%) ^△^				
≤45	43(56.6)	18(64.3)	1.700	0.427
45–60	20(26.3)	4(14.3)		
>60	13(17.1)	6(21.4)		
Expression of P53 ^△^				
Negative	28(46.7)	15(57.7)	0.882	0.348
Positive	32(53.3)	11(42.3)		
Expression of P16 ^△^				
Negative	38(64.4)	16(72.7)	0.499	0.480
Positive	21(35.6)	6(27.3)		
TNM Stage ^△^				
I/II	42(62.7)	21(77.8)	1.983	0.159
III/IV	25(37.3)	6(22.2)		
Lymph node metastasis ^△^				
Positive	20(33.9)	11(47.8)	1.365	0.243
Negative	39(66.1)	12(52.2)		

^△^ Some patients were excluded from the analysis if this clinical parameter was missing. * Value obtained by Fisher’s exact probability method.

## Data Availability

The original contributions presented in this study are included in the article/Supplementary Materials. Further inquiries can be directed to the corresponding authors.

## Supplementary Materials

The following supporting information can be downloaded at: https://www.mdpi.com/article/10.3390/ijms262411772/s1.

## Abbreviations

The following abbreviations are used in this manuscript:

OSCC	oral squamous cell carcinoma
MAGE	melanoma-associated antigen
OLK	oral leukoplakia
TCGA	The Cancer Genome Atlas
IHC	immunohistochemistry
OS	overall survival
DFI	disease-free interval
DSS	disease-specific survival
PFI	progression-free interval
GO	gene ontology
DAC	5-aza-2′-deoxycytidine
TSA	trichostatin A
VPA	valproic acid
RT-PCR	reverse transcription polymerase chain reaction
qRT-PCR	quantitative reverse transcription polymerase chain reaction
DNMT	DNA methyltransferase
ChIP-qPCR	chromatin immunoprecipitation-qPCR
HLA	human leukocyte antigen
CTL	cytotoxic T lymphocyte
HDAC	histone deacetylase
MBD	methyl-CpG binding domain
HNSC	head and neck squamous cell carcinoma
DMEM	Dulbecco’s modified Eagle medium
FBS	fetal bovine serum
SAM	s-adenosylmethionine
RT	room temperature
