# Supplementary material for: Intratumoral Heterogeneity of MAGED4 Expression in Oral Squamous Cell Carcinoma: Epigenetic Mechanisms and Therapeutic Implications
Source: Int J Mol Sci. 2025 Dec 5;26(24):11772. doi: 10.3390/ijms262411772 (PMC12732915; doi:10.3390/ijms262411772)
Supplement: Supplementary file 1 [file ijms-26-11772-s001.zip › ijms-3970673-supplementary.pdf]

International Journal of
Molecular Sciences

MDPI

Supplementary Materials

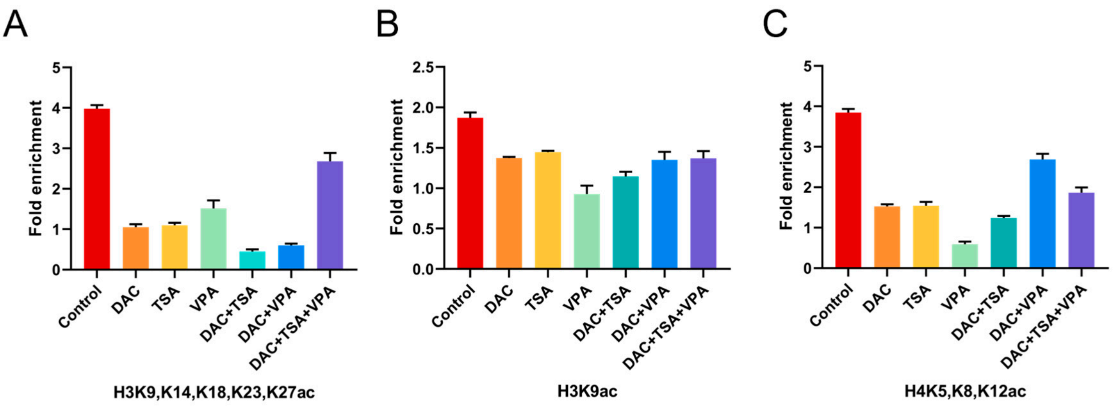

Figure S1. ChIP-qPCR analysis of Histone Acetylation at the MAGE-D4 promoter in CAL-27. (**A**) Enrichment of Acetylated H3 Histone Marks （K9, K14, K18, K23, and K27）; (**B**) H3K9 Acetylation-Specific Enrichment; (**C**) Enrichment of Acetylated H4 Histone Marks (K5, K8, and K12)

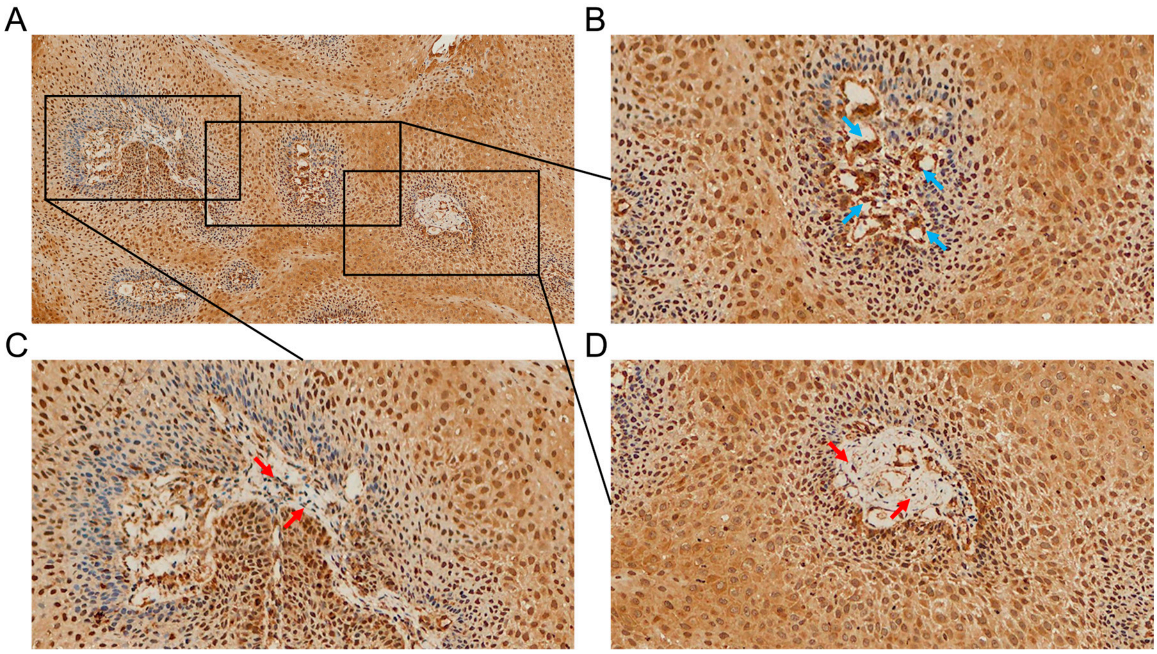

Figure S2. MAGED4 expression in endothelial cells and fibroblasts within OSCC Tissues. (A) OSCC tissue with high MAGED4 expression. The MAGED4 protein is predominantly localized in the cytoplasm and nucleus of cancer cells. (B) Endothelial cells in OSCC tissues, showing MAGED4 expression. Endothelial cells were marked with blue arrows. (C-D) Fibroblasts in OSCC tissues. Fibroblasts were marked with red arrows.
